# Ex Vivo MRI Analytical Methods and Brain Pathology in Preterm Lambs Treated with Postnatal Dexamethasone †

**DOI:** 10.3390/brainsci10040211

**Published:** 2020-04-03

**Authors:** Nathanael J. Yates, Kirk W. Feindel, Andrew Mehnert, Richard Beare, Sophia Quick, Dominique Blache, J. Jane Pillow, Rod W. Hunt

**Affiliations:** 1 School of Human Sciences, University of Western Australia, Perth 6009, Australia; jane.pillow@uwa.edu.au; 2 Queensland Brain Institute, University of Queensland, Brisbane 4072, Australia; 3 Centre for Microscopy, Characterisation and Analysis, University of Western Australia, Perth 6009, Australia; kirk.feindel@uwa.edu.au (K.W.F.); andrew.mehnert@uwa.edu.au (A.M.); shmwardell@gmail.com (S.Q.); 4 School of Biomedical Sciences, University of Western Australia, Perth 6009, Australia; 5 Developmental Imaging, Murdoch Children’s Research Institute, Melbourne 3052, Australia; richard.beare@mcri.edu.au; 6 Department of Medicine, Monash University, Melbourne 3800, Australia; 7 School of Agriculture and Environment, University of Western Australia, Perth 6009, Australia; dominique.blache@uwa.edu.au; 8 Murdoch Children’s Research Institute, Melbourne 3052, Australia; rod.hunt@rch.org.au; 9 Department of Paediatrics, University of Melbourne, Melbourne 3052, Australia

**Keywords:** animals, neonatal, neurodevelopmental disorders, infant, premature, magnetic resonance imaging, neuropathology, neurology, glucocorticoids, dexamethasone

## Abstract

Postnatal glucocorticoids such as dexamethasone are effective in promoting lung development in preterm infants, but are prescribed cautiously due to concerns of neurological harm. We developed an analysis pipeline for post-mortem magnetic resonance imaging (MRI) to assess brain development and hence the neurological safety profile of postnatal dexamethasone in preterm lambs. Lambs were delivered via caesarean section at 129 days’ (d) gestation (full term ≈ 150 d) with saline-vehicle control (Saline, *n* = 9), low-dose tapered dexamethasone (cumulative dose = 0.75 mg/kg, *n* = 8), or high-dose tapered dexamethasone (cumulative dose = 2.67 mg/kg, *n* = 8), for seven days. Naïve fetal lambs (136 d gestation) were used as end-point maturation controls. The left-brain hemispheres were immersion-fixed in 10 % formalin (24 h), followed by paraformaldehyde (>6 months). Image sequences were empirically optimized for T1- and T2-weighted MRI and analysed using accessible methods. Spontaneous lesions detected in the white matter of the frontal cortex, temporo-parietal cortex, occipital lobe, and deep to the parahippocampal gyrus were confirmed with histology. Neither postnatal dexamethasone treatment nor gestation showed any associations with lesion incidence, frontal cortex (total, white, or grey matter) or hippocampal volume (all *p* > 0.05). Postnatal dexamethasone did not appear to adversely affect neurodevelopment. Our post-mortem MRI analysis pipeline is suitable for other animal models of brain development.

## 1. Introduction

Immaturity of the lung and prolonged mechanical ventilation is a risk factor for the development of chronic lung disease in preterm infants. Mechanical ventilation of preterm infants is also associated with brain injury, especially used over a prolonged period and when excessive tidal volumes are used [1]. Consequently, early extubation and the use of non-invasive respiratory support is a key goal of contemporary neonatal clinical practice. Postnatal glucocorticoids, such as dexamethasone, promote lung development and facilitate extubation in infants with severe lung disease [2,3], but are prescribed cautiously, because of concerns of increased risk for poor neurodevelopment [3]. 

The supraphysiological doses of dexamethasone administered to preterm infants two decades ago reduced cortical grey matter, cerebral volume and cerebellar volume at term-equivalent age [4,5]; and resulted in smaller hippocampi at 2 years’ corrected postnatal age [6]. Some of these changes persist into adolescence [7]. Postnatal high dose dexamethasone therapy in preterm infants is also associated with trends for increased neuropathology, such as periventricular leukomalacia (i.e., deep cystic and diffuse white matter lesions) and intraventricular haemorrhage [8,9]. However, several volumetric changes were not replicated [10]. Furthermore, meta-regression analyses show that postnatal dexamethasone may be beneficial for neurodevelopment in infants at high risk of chronic lung disease, and harmful for infants at low risk, independent of dose [11,12]. 

Contemporary postnatal dexamethasone protocols use a tapered low-dose approach to reduce the risk of neurological harm: low-dose postnatal dexamethasone is efficacious for improved lung outcomes, but current studies are underpowered to detect potential adverse neurodevelopmental outcomes [13,14]. The lack of clarity on the role of contemporary low-dose postnatal dexamethasone dosing on brain development remains concerning for clinicians and parents. 

Clinical imaging of brains collected from preterm animals during preclinical studies may provide insight into the dose-dependent effects of postnatal dexamethasone. However, characterization of magnetic resonance imaging (MRI) pathology with histology is often difficult, as clinical-quality in vivo MRI scans (high resolution and contrast) are often not generated from large animals. Furthermore, automated segmentation of white and grey matter is difficult in immature brains with incomplete myelination. Ex vivo imaging has potential advantages over in vivo imaging, such as the ability to have longer imaging-sessions (thus higher resolution), ability to correlate imaging with histology, and use of archival tissue. However, the effects of fixation mean that new sequences are needed to generate MR images with clinical-quality contrast and new methods are needed to assist segmentation and analysis.

We aimed to develop a workflow pipeline for the ex vivo examination of preterm lamb brains, and to use the resulting MRI derived tissue volumes to examine the effects of early postnatal glucocorticoid therapy on brain growth and the development of neuropathology. Specifically, we aimed to use adaptive and intuitive tissue segmentation techniques to overcome limitations associated with ex vivo imaging, by establishing MRI sequence scan parameters that would result in image appearances similar to those obtained on clinical MRI scans.

## 2. Materials and Methods

### 2.1. Animal Studies

All experiments were approved by the University of Western Australia (UWA) Animal Ethics Committee (Approval number: RA/3/100/1301) and followed all relevant local and national guidelines for experimental animal use. 

Date-mated ewes received intramuscular medroxyprogesterone (150 mg/mL, Pfizer Australia, Sydney, Australia) one week prior to intended delivery date, to prevent spontaneous birth following intramuscular betamethasone injections (5.7 mg, Merck Sharp & Dohme Pty Ltd., Macquarie Park, Sydney, Australia), administered 48 h and 24 h prior to delivery. Preterm lambs were delivered via hysterotomy at 129 days’ (d) gestation (full term ≈ 150 d) and then assigned to one of three experimental groups: saline vehicle control (Saline, *n* = 9), low-dose tapered dexamethasone (Low Dex, *n* = 8), or high-dose tapered dexamethasone (High Dex, *n* = 8) for 7 days. Low dose dexamethasone comprised 0.15 mg/kg for 3 days, 0.1 mg/kg for 2 days and 0.05 mg/kg for 2 days, with a total cumulative dose of 0.75 mg/kg. A high dose dexamethasone comprised 0.5 mg/kg for 3 days, 0.3 mg/kg for 3 days and 0.27 mg/kg for 1 day, with a cumulative dose of 2.67 mg/kg. The maturational age was chosen due to relative lung immaturity, sensitivity to ventilator injury, and consequently, relevance to clinical postnatal glucocorticoid treatment. The saline group was exposed to the same postnatal environment and conditions, however received equal volume saline injections over the 7-day period. All three preterm lamb groups were managed according to routine contemporary neonatal protocols including surfactant, using a progressive de-escalation of respiratory support and extubation to non-invasive support as soon as possible. Naïve fetal control lambs (no postnatal life, no antenatal medroxyprogesterone or betamethasone exposure, *n* = 7) were delivered by hysterotomy at 136 d gestation as an end-point maturational reference for naïve (normal) fetal brain development.

### 2.2. Brain Preparation for MRI

All lambs were euthanized at 136 d postconceptional age with an intravenous pentobarbitone overdose (150 mg/kg). The lamb brains were dissected rapidly and divided into left and right hemispheres. The right hemispheres were frozen for use in other studies. Left hemispheres were fixed (24 h, 10% neutral buffered formalin, Sigma Aldrich, Castle Hill, Australia), then immersed in 4 % paraformaldehyde (Sigma Aldrich) for 6–24 months. Data from mouse MRI studies suggest that long-term storage modifies brain volumes, but that this may plateau after approximately 5 months [15]. Brains were placed in phosphate-buffered saline (PBS) with 0.1% sodium azide, for a minimum of one week prior to imaging. 

**Figure 1:**
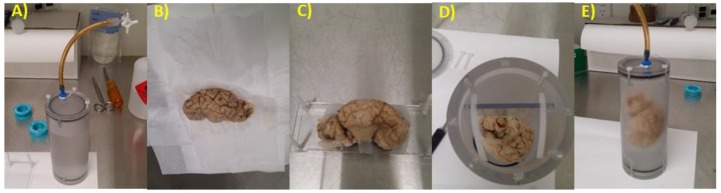
Brain tissue preparation for MRI acquisition. (**A**) The custom-made chamber has a tight seal which is secured with plastic screws. The lid has a tube attached to a 3-way tap to allow de-gassing. (**B**) The brain is patted dry with Kimwipes^®^ (Kimberly-Clark), until no more water is present on the tissue. (**C**) The brain is gently taped onto the platform, so that it will not move. (**D**) The platform should be a secure fit in the chamber. (**E**) The lid is then placed back on securely, and the brain is fully immersed in Fluorinert, and de-gassing can commence.

Brains were scanned in a custom-built MRI chamber (Figure 1A). The brains were removed from phosphate buffered saline (PBS), and patted until the surface was dry to the touch (Figure 1B). The brain hemisphere was gently taped to a rectangular platform, which neatly fits into the custom-made scanning chamber (Figure 1C,D). The brain was then immersed in a non-protonated, susceptibility matched fluid (Fluorinert^TM^ FC770, 3M Co., St. Paul, MN, USA) and sealed in the chamber (Figure 1E). The brain was degassed then re-pressurized to ambient pressure. This degassing process was repeated until no further bubbles were removed. Chambers were left overnight or longer to allow additional gas to dissolve in the solution.

### 2.3. Image Sequence Development

**Figure 2:**
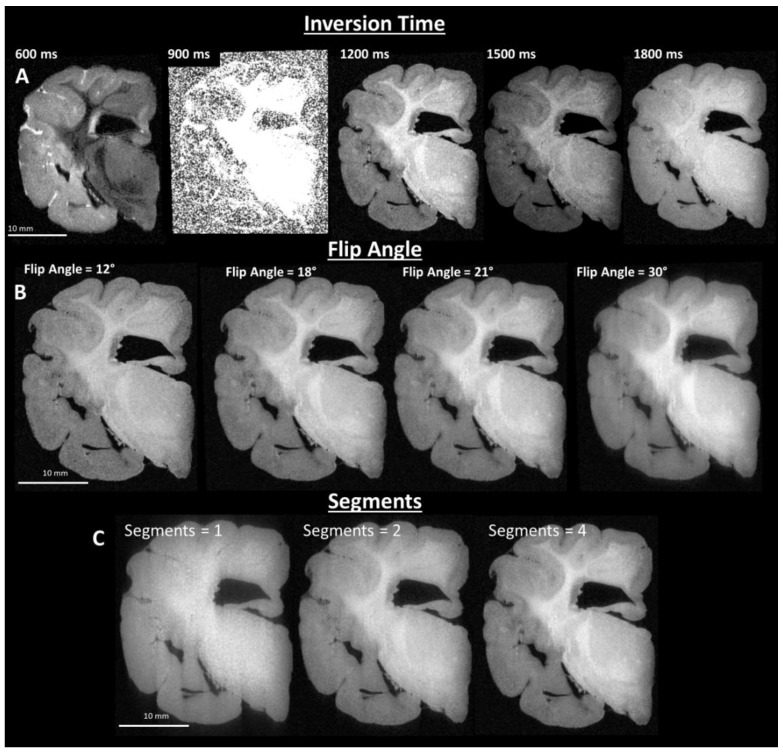
Imaging parameter optimization of T1-weighted images. (**A**) Inversion time optimization results in optimal image contrast between grey matter and white matter, by systematically varying the time delay between inversion of the MRI signal and signal acquisition (top left corners of image). (**B**) Imaging flip angle optimization results in adjustment of the image signal-to-noise and contrast by systematically varying flip angle. (**C**) Imaging sequence optimization of segmentation is varied to maintain the T1 weighted contrast produced by the initial signal inversion and delay to signal acquisition, collection of the data is segmented, e.g., for one segment a single inversion pulse is followed by signal acquisition in one 2D plane of k-space, whereas for four segments, following each inversion pulse, one-quarter of a 2D plane is acquired, sequentially.

Images of post-fixed brains were acquired using a 9.4 T preclinical MRI system (Bruker BioSpec, Billerica, MA, USA), with a Bruker 94/30 US/R superconducting magnet, Avance III HD console, BGA-12SHP imaging gradients, and 72 mm quadrature transmit/receive volume coil, with ParaVision 6.0.1 software (Bruker, Billerica, MA, USA). T1 weighted images were acquired with the Bruker MDEFT method, operating as an inversion recovery (IR) prepared segmented 3D fast low angle shot (FLASH) imaging sequence. Acquisition parameters were optimized empirically to provide image contrast between grey matter and white matter that was visually similar to the contrast obtained with in vivo brain tissue. Final sequence parameters were: 4 segments, 4 s segment repetition time (TR), 1200 ms inversion recovery delay, 12 ms echo repetition time, 21° flip angle (α), 5 ms echo time (TE), and 8 averages. T2 weighted images were acquired with the Bruker TurboRARE method. Acquisition parameters were optimized empirically to provide image contrast between grey matter and white matter that was visually similar to contrast obtained with in vivo brain tissue. Final sequence parameters were: TR = 1.8 s, 12 echoes, 10 ms echo spacing with 30 ms effective TE, 90° flip angle (α), and 4 averages. The size of the 3D image matrix was adjusted based on brain size to provide a consistently isotropic voxel size (120 μm)^3^ for T1-weighted images, and (150 µm)^3^ for T2-weighted images. T1-weighted image sequence parameter optimization results are shown in Figure 2A–C. 

### 2.4. Grey and White Matter Tissue Segmentation

Ex vivo MRI scanning on post-fixed preterm lamb brain tissue presents several unique challenges: variable tissue fixation quality and duration, susceptibility artefacts due to gas bubbles, absence of a standardized brain atlas for this gestational period, poor tissue contrast due to incomplete myelination, and deformation of brains post-fixation. Additionally, our MRIs were limited to the left hemisphere. Several strategies were initially explored to segment white and grey matter interactively using ITK-SNAP [16], and automatically using FSL FAST [17,18]. However, neither strategy proved satisfactory.

We instead developed an interactive image analysis workflow to facilitate the accurate segmentation and measurement of ex vivo images that have large qualitative differences between specimens, utilizing a suite of open source tools available in the Characterisation Virtual Laboratory desktop (https://www.cvl.org.au/). These tools include: 3D Slicer, FreeSurfer, and MRIcroGL. This resource is a remote Linux desktop environment, providing Australian researchers with access to high performance computing using a range of software tools, including neuroimaging, to analyse imaging and characterization data. 

#### 2.4.1. Pre-Processing

**Figure 3:**
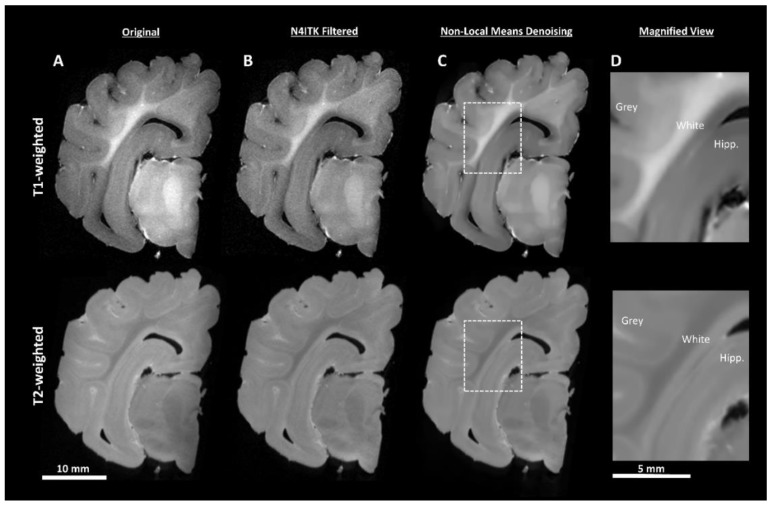
Image pre-processing demonstrated by T1- and T2-weighted coronal sections (**A**). N4ITK bias field correction (**B**) removes bright and dark patches in the image resulting in more uniform tissue-type intensity. The use of non-local means denoising (**C**) attenuates the high-frequency noise in the image, creating more uniform tissue boundaries. A magnified view of the boxed regions is shown in (**D**) encapsulating part of the cortex (White and Grey) and hippocampus (Hipp.) All images within each have normalized histograms to allow for quality comparisons.

DICOM (https://www.dicomstandard.org/) files were batch converted to the NIfTI format (https://nifti.nimh.nih.gov/) using MRIcroGL (https://www.mccauslandcenter.sc.edu/mricrogl/). MRI images often exhibit a low-frequency intensity variation known as the bias field (Figure 3A). The N4ITK [19] module in 3D Slicer (Version 4.80, https://www.slicer.org/) [20] was used to perform bias field correction (Figure 3B). Noise attenuation was performed using a MATLAB implementation [21] of the non-local means algorithm (Figure 3C), which uses adaptive 3D averaging based upon voxel similarity and not only geometric proximity [22]. At this stage it was apparent that the T1-weighted sequence demonstrated better white/grey matter tissue contrast, showed more consistent image appearance, and was better able to delineate the hippocampus from surrounding tissue than the T2-weighted sequence (Figure 3D). Therefore, only T1-weighted images underwent further processing for segmentation. Finally, the images were manually re-orientated to a common alignment plane using FreeSurfer (version 6.0) [23], to ensure that anatomical orientation labels were correct.

#### 2.4.2. Segmentation of the Cortical Structures and Hippocampus

Manual segmentation of the cortical structures was based upon published MRI sheep brain atlases [24,25]. The hippocampus was defined based upon the Michigan State sheep brain atlas [26] and was determined by a detailed examination in each of the orthogonal imaging planes.

**Figure 4:**
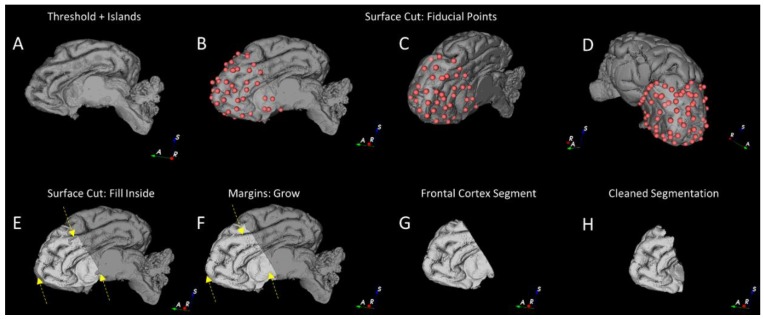
Example segmentation of the frontal cortex using Slicer 3D workflow. (**A**) The 3D MRI volume generated in Segment Editor has the functions “Threshold -> Use for masking”, followed by “Islands -> Keep largest island”. (**B**–**D**) Fiducial markers are placed on the rendered volume based upon surface features defined from an MRI atlas. The tool used is “Surface Cut -> Fiducial Placement -> Set”. (**E**) The rough frontal cortex segment is generated using the “Surface Cut -> Fill Inside” setting, which leaves some small surface regions unlabelled (yellow arrows). (**F**) Using the “Margin -> Grow” tool, the incomplete surface labels are improved (yellow arrows). (**G**) The rough frontal cortex can be extracted at this stage. (**H**) The gross segmentation can be cleaned using the “Paint” and “Erase” tool with the “Sphere brush” setting, followed by liberal use of the “Smoothing” to generate a refined segmentation, which matches the regions defined in atlas of choice.

Initial gross segmentation of regions of interest was performed using the “Segment Editor” module of 3D Slicer (4.80) and the extensions “SegmentEditorExtraEffects”, “SegmentationWizard”, and “MarkupsToModel”. Initial segmentation of the whole brain mask was performed using the “Threshold” and “Islands” tools, followed by manual clean-up with the “Paint” tool (Figure 4A).

Briefly, we defined the frontal cortex by the areas defined as 1, 4, 13, 14, 15, 19, and 22 in Ella et al. 2017 [24]. This region was chosen because the tissue boundaries were readily discernible and it was not significantly impacted by scanning artefacts due to undissolved gas (in contrast to the lateral ventricles). Using the 3D rendered image of the masked brain volume, we placed fiducial points on the brain volume surface based upon landmarks in the Ella et al. atlas [24] with the “Surface Cut” (Figure 4B–D) tool and filled this region (Figure 4E). Final editing was performed with “Margins Grow”, “Paint” and “Smoothing” on individual image slices (Figure 4F–H). More detailed methods can be found in the Supplementary Materials.

The hippocampus was segmented manually using “Segment Editor” in 3D Slicer, based upon closely examining slice planes and the 3D volume render. The segmentation was performed using a combination of the “Surface Cut” tool in the coronal plane, followed by editing with the “Paint” and “Smoothing” tool. The hippocampus was not further segmented into tissue types.

#### 2.4.3. Segmentation of the White and Grey Matter

**Figure 5:**
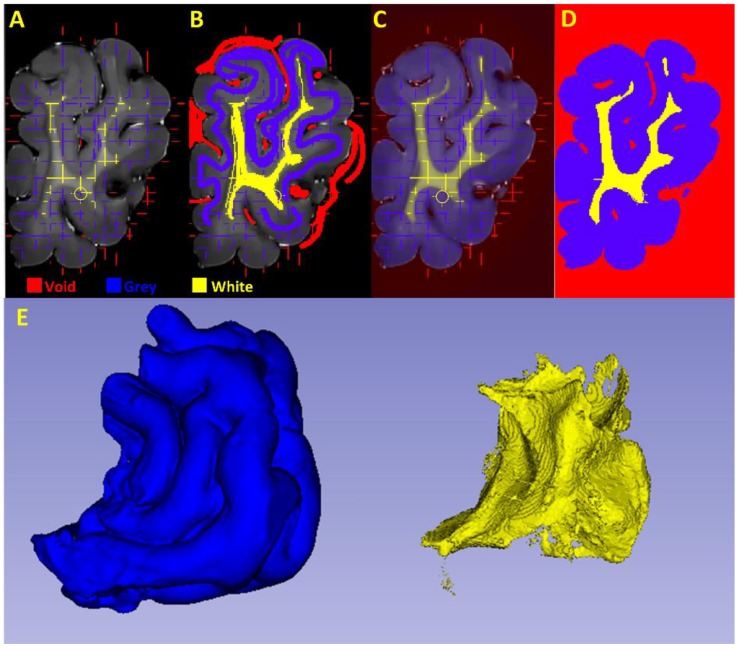
Segmentation of the frontal cortex in a T1-weighted image. Segmentation used supervised machine learning in ilastik. Tissue labels “Void”, “Grey matter” or “White matter” are coloured red, blue and yellow respectively. (**A**) Original coronal T1-weighted MRI slices, with labelled segments in orthogonal planes indicated by coloured lines. (**B**) Painted segmentation for training in the current plane. (**C**) Probability map of tissue classification, and (**D**) the final tissue segmentation used for quantification. The final 3D volume renderings generated in 3D slicer can be seen in (**E**).

The frontal cortex files were converted to HDF5 format using FIJI [27]. White and grey matter segmentation was then performed using the “Pixel Classification” workflow in ilastik (version 1.2.2, https://www.ilastik.org/) [28] with all features selected for supervised learning. Three classes (labels) were defined for training: “Void”, “Grey Matter”, and “White Matter”. “Void” was defined as a catch-all class for regions without tissue and/or regions containing imaging artefacts. Regions for each class were painted interactively (supervised learning) using the three orthogonal planes, until a satisfactory segmentation was achieved using “Live Update” mode: this mode permits the segmentation accuracy to be monitored (prediction probabilities and segmentation). Figure 5 shows the frontal cortex image in the coronal plane (Figure 5A), the training labelling (Figure 5B), probability maps generated from the supervised machine learning (Figure 5C), the final tissue segmentation (Figure 5D) and resulting segmented 3D volume (Figure 5E). Volumetric analysis was performed in FIJI using custom-written macros, which take into account the equivalent volume of each voxel size in the MRI and final number of voxels in each tissue class for the final HDF5 exported image (ImageJ version 1.52h, [27]).

#### 2.4.4. Gross Anatomical Measurements

Each T1 scan was scored by two examiners (RWH and NJY) for signs of pathology, such as deep white matter lesions, signal abnormalities or cysts using MRView (The MRtrix viewer, version 3.0, 64-bit version, https://www.mrtrix.org/) [29]. Each examiner was blinded to the treatment group until after scoring was complete. Pathology was decided based upon consensus. All T2-weighted scans were also examined blinded to group (NJY). If pathology was detected in either T1- or T2-weighted images, they were viewed side-by-side in 3D slicer to confirm lesion.

### 2.5. Histology

Selected blocks of brain tissue were cut to examine the putative lesion sites, based upon MRI observations. Blocks were paraffin embedded, sectioned at 5 µm thickness on a microtome (Leica Microsystems, Mt Waverley, Australia, RM2255), and then placed upon slides (Superfrost^®^ Plus, Thermo Scientific, Scoresby, Australia, Cat: MENSF41296SP). Tissue sections were stained with hematoxylin and eosin (H&E) using established protocols (Harris’ Hematoxylin and 1% Eosin Y solutions), or were processed for peroxidase immunohistochemistry. 

### 2.6. Immunohistochemistry

Tissues for immunohistochemistry were dewaxed in 2 × 100% Toluene, rehydrated in 100% ethanol, 70% ethanol, and then rinsed in tap water. Sections were washed twice with PBS, incubated in 3% H_2_O_2_ in PBS for 20 min to deactivate endogenous peroxidase, followed by an additional two PBS washes. Sections were then incubated in blocking solution for 30 min (5% goat serum, 0.2% triton X-100, in PBS). The tissue was incubated overnight in primary antibodies raised against ionized calcium binding adaptor molecule 1 (IBA1) for microglia staining (1:500, Wako, Species: Mouse, Cat: 019-19741), glial fibrillary acidic protein (GFAP) for astrocyte staining (1:1000, Dako, Species: Rabbit, Cat: Z0334), caspase-3 for apoptosis (1:1000, R&D Systems, Species: Rabbit, Cat: AF835), and myelin basic protein (MBP) for myelin staining (1:500, Millipore, Species: Rat, Cat: MAB386), and all primary antibodies were made blocking solution at 4 °C. Tissue stained for IBA1 and caspase-3 had an additional antigen retrieval step prior to the H_2_O_2_ incubation (0.01 M Citrate Buffer Solution, pH = 6, 5 min at 98 °C, H2500 Microwave processor, Bio-Rad, Gladesville, Australia).

Tissues underwent PBS washes in triplicate the following day, followed by incubation in appropriate species-specific biotinylated secondary antibodies (1:200, anti-rabbit IgG, Cat: BA-1000, anti-mouse IgG, Cat: BA-2000, anti-rat IgG, Cat: BA-9400; Vector Laboratories) for two hours. Sections were stained using the Vectastain Elite Avidin-Biotin Complex kit (1:200 reagent A, 1:200 reagent B, 90 min incubation at room temperature; PK-4002, Vector Laboratories, Burlingame, CA, USA). The antibody complex was visualized using 3,3′-diaminobenzidine—horseradish (DAB) peroxidase reaction (1 tablet of DAB and 1 tablet of urea per 10 mL, Sigma-Aldrich Co., St Louis, MI, USA, 10–20 min). Each reaction was stopped with three PBS washes. The tissue was then processed with ascending concentrations of ethanol, cleared in toluene and then cover-slipped in Micromount^®^ mounting media (Leica Microsystems, Cat: 3801731). All slides were scanned at 20 × magnification (Aperio ScanScopeXT, Leica Microsystems), and the images were examined in ObjectiveView^TM^ (version 1.48, Halton Hills, Ontario, Canada) and exported to FIJI for further analysis (ImageJ version 1.52h, [27]).

### 2.7. Data Analysis

An unpaired *t* test was used to test for significant differences between the fetal control group and the preterm saline group. Variances were compared with F-test. One-way ANOVAs were used to compare outcomes across the three postnatal preterm groups (Saline, Low-Dex, and High-Dex) with Brown-Forsythe test used to compare standard deviations. Kruskal-Wallis tests were used when assumptions required for ANOVAs were not met. Post-hoc tests were planned using Dunnett multiple-comparisons. Statistical analysis and graphs were generated using GraphPad Prism (version 7.04, GraphPad Software Inc., San Diego, CA, USA) All tests were performed at α = 0.05 level of significance. The data that support the findings of this study are available from the corresponding author upon reasonable request. 

## 3. Results

### 3.1. Clinical Variables

A summary of clinical variables and associated inference tests is shown in Table 1. Briefly, the results provide evidence that prematurity affected reduced birth weight (*p* = 0.0002), weight at equivalent conception age (*p* < 0.0001) and post-mortem brain weight (*p* = 0.0369). The results also provide evidence that dexamethasone therapy had no effect on any clinical variable (all *p* > 0.05). For all reported measures below, we also examined whether tissue age at time of scanning had an effect on outcome measures in the preterm animals. There was no correlation between age of tissue on any outcome measure (all *p* > 0.05).

### 3.2. Magnetic Resonance Imaging

#### 3.2.1. Volumetric measures

**Figure 6:**
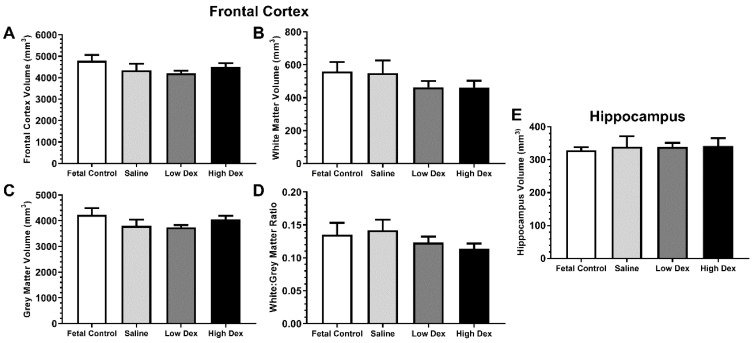
MRI volumetric measurements of the frontal cortex and hippocampus. Frontal cortex (**A**) total volume, (**B**) white matter volume, (**C**) grey matter volume, (**D**) white:grey matter ratio. (**E**) Hippocampus total volume. Bars represent mean + SEM. No significant differences between fetal control and saline, or between preterm groups (all *p* > 0.05).

Table 2 summarizes the results of inference tests for the volumetric measures obtained from MRI. The results provide evidence that frontal cortex volumes were unchanged by prematurity and dexamethasone therapy, including total volume (Figure 6A), white matter volume (Figure 6B), grey matter volume (Figure 6C), and white to grey matter ratio (Figure 6D). The results also provide evidence that hippocampal volume was also unchanged by prematurity or postnatal dexamethasone (Figure 6E).

#### 3.2.2. Anatomical Measures

Table 3 summarizes the results of inference tests for anatomical measures of the brain gross morphology. The results provide evidence that the dimensions were not influenced by preterm birth and seven days of postnatal care (Table 3; *t*-tests, all *p* > 0.05), or by postnatal dexamethasone treatment (ANOVA, all *p* > 0.05).

### 3.3. Neuropathology

**Figure 7:**
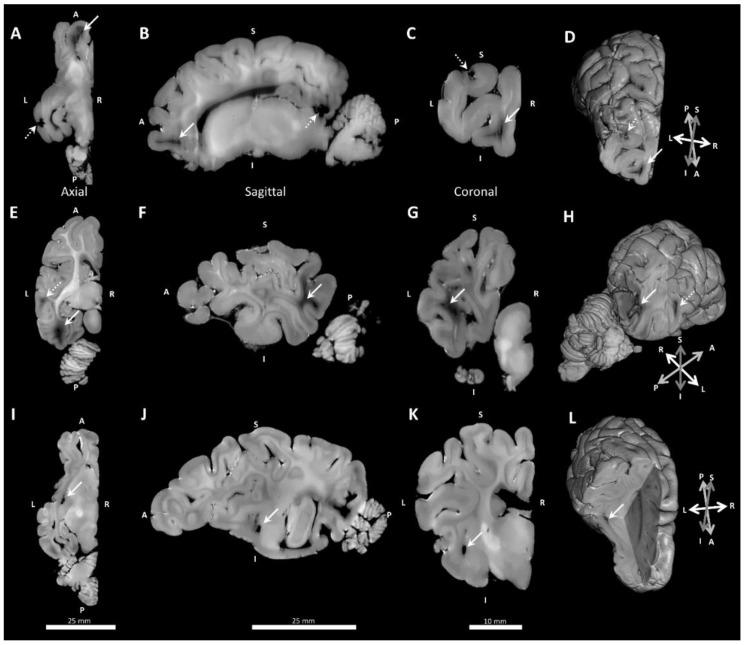
Example lesions in T1-weighted MRI images shown in orthogonal planes and with 3D rendering. (**A**–**D**) Frontal cortex white matter lesions (solid white arrows) and examples of susceptibility artefacts due to undissolved gas (stippled arrows). (**E**–**H**) Temporal lobe cortex lesion (stippled arrow) and occipital cortex white matter lesion (solid white arrow). (**I**–**L**) Deep cystic lesions (solid white arrows). Abbreviations: L, Left; R, Right; I, Inferior; S, Superior; A, Anterior; P, Posterior.

Two main types of white matter pathology were observed in MRIs: 1) white matter lesions in the frontal cortex (Figure 7A–D), temporo-parietal and occipital cortex (Figure 7E–H); and 2) cystic dissolution in the lateral boundary anterior to the amygdala and medial to the parahippocampal gyrus (Figure 7I–L). Cystic lesions were differentiated from large deeply penetrating blood vessels based upon size and shape, and were confirmed with histology in the two control fetal lambs that had MRI evidence of cystic lesions (Table 4). Independent analysis of T1- and T2-weighted images showed that the same white matter lesions were detected using either imaging sequence, except in one case, where T2-weighted image missed a frontal cortex lesion due to trapped air, appearing to be similar to white matter on initial inspection. 

**Figure 8:**
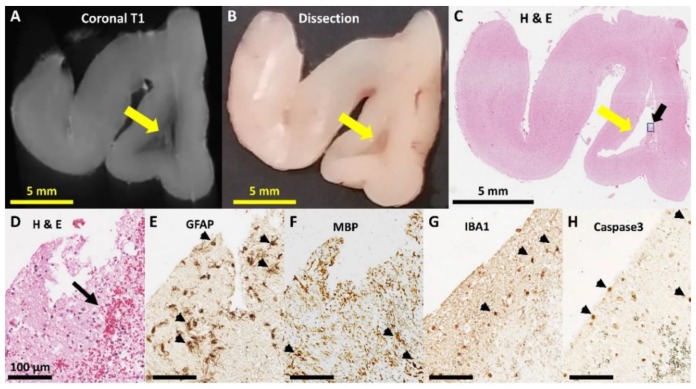
Frontal cortex lesion examined using MRI, dissection, histology and immunohistochemistry. The large white matter lesion (yellow arrows) are observed in the coronal plane of the prefrontal cortex with (**A**) T1-weighted MRI image, (**B**) dissection, and (**C**) H&E staining. Close examination of the lesion indicated by boxed region and black arrow in (**C**) reveals red blood cells with H&E staining in the lesions site (arrows in (**D**), local changes in GFAP+ astrocyte morphology (arrows in (**E**)), fragmentation of myelin (throughout tissue) and intense oligodendrocyte staining with MBP (arrows (**F**)), intense local microglia staining (arrows in (**G**)), and localized apoptosis indicated with Caspase 3 staining (arrows in (**H**)).

An example of anatomical lesion is shown Figure 8. T1-weighted MRI slices (Figure 8A) were matched anatomically with photographic (Figure 8B) and low-magnification images of brain tissue (Figure 8C), that were examined subsequently at higher resolution (Figure 8D–H) to confirm the presence of histopathology. A range of histopathological lesions were identified in brain sections. Briefly, confirmed lesion sites often had localized deposits of red blood cells observed in H&E staining (Figure 8D), highly branched and intensely-stained astrocytes (GFAP, Figure 8E), fragmented white matter tracts and intensely-labelled oligodendrocytes (MBP, Figure 8F), intensely stained microglia with a reactive rounded morphology (IBA1, Figure 8G), and patches of apoptotic cells (Caspase-3, Figure 8H). These patterns of staining were not observed in locations distal to the lesion site or in animals that did not have MRI-detectable signs of lesions.

## 4. Discussion

We established MRI sequence parameters and developed interactive image analysis workflows suitable for the ex vivo imaging of fixed preterm lamb brains. Our methods enabled segmentation of brain tissue into white and grey matter, and the detection of spontaneous lesions in multiple brain regions, subsequently confirmed with histology. We applied this workflow to examine the effect of postnatal dexamethasone on anatomical and volumetric indices of brain development and integrity. There were no significant differences in anatomical or volumetric indices of brain development attributable to postnatal dexamethasone therapy.

The use of high field MRI scans with parameters empirically optimized for ex vivo samples achieved excellent tissue contrast and much higher resolution MRI than shown in previous in vivo [24,25,30] and ex vivo sheep studies [24,25]. The quality of the MRIs allowed detection of lesions that were not evident on gross visual inspection of the brains, and which would be missed otherwise. Our study would have benefitted from diffusion tensor imaging measurements to examine axonal integrity and connectivity, similar to previous studies in ventilated preterm lambs [30]. However, pilot testing for diffusion tensor imaging (DTI) proved difficult in our post-mortem tissue, requiring excessive scanning time and additional optimization to be usable. Signal contrast on DTI relies on the detection of differential diffusion of water molecules through living tissue affected by oedema, inflammation or maturational variation. Theoretically, this was not worth pursuing in the post-mortem model, and the interpretation of findings, even with the benefit of histology, would have been challenging and almost certainly not informative.

Premature animals, much like human infants, have reduced myelination and thus poorer tissue contrast in MRI requiring specialized methods for segmentation [31]. Furthermore, ex vivo brain imaging has challenges due to variable tissue fixation, and changes in water diffusivity compared to in vivo imaging [32,33]. However, we overcame these challenges using our tissue preparation, MRI sequence optimization, and interactive image analysis workflow. We achieved excellent tissue segmentation and visualization, establishing methods which will be easy to implement in future studies. Our workflow allowed us to measure brain regional volumes, localize lesions, and correlate lesions with histopathology. The use of open source software also facilitates the future use of this workflow. The quality of imaging would likely be enhanced by perfusion-fixation. However, the success of our imaging and analytical methods in our study demonstrate potential use of our methods in diverse types of archival and developmentally immature preserved tissue.

The use of postnatal dexamethasone to promote lung development in preterm infants is contentious. Strong recommendations not to use postnatal steroids due to neurological risks in preterm infants to promote or protect lung development [34] have persisting influence, despite a more nuanced picture emerging from the most recent systematic reviews [3,35]. Our study suggests that 7 days of postnatal dexamethasone administered at low and high doses do not have obvious short-term effects on brain growth or damage in preterm lambs when contemporary lung-protective respiratory management strategies are used. This finding must be interpreted with caution, as the small numbers of animals in each group preclude the conclusion that dexamethasone is not injurious to the developing brain, nor do our findings preclude the possibility of long-term changes in brain growth or development: changes in brain volumes in preterm adolescents exposed to postnatal dexamethasone [7] are more robust than changes observed in infants at near term-equivalent postnatal age [4,10]. Other brain injury models in sheep show that one week following any potentially adverse exposure may be an early time-point to observe large structural changes. For example, fetal hypoxia-ischemia in 0.65 gestation fetal sheep reduces brain weight and white matter volume at two weeks, but not at one week post-injury [36]. Furthermore, the brains of 129 d gestation lambs used in the current study are more comparable to maturity of the near-term brain in the human infant [37], and are in relatively late stages of myelination [38]. Thus, the 129 d gestation lamb is probably less vulnerable to the adverse effects of dexamethasone on brain development than the typical preterm human infant at 0.8 gestation. For example, we did not find any clear evidence of T2-weighted hypo- and hyper-intensities, described previously in hypoxic-ischemic lambs at earlier gestation (0.65 gestation injury) [38].

Despite a relative maturity of the preterm lamb brain at 129 d gestation (near-term human equivalent), the 129 d preterm lamb brain is not immune to harm from mechanical ventilation. High tidal volume injurious ventilation in preterm lambs of similar gestation induces brain injury observable in gross histology, elevated lipid peroxidation and vascular extravasation, compared to unventilated controls [39]. Protective ventilation reduces periventricular lipid peroxidation and vascular extravasation, compared to high tidal volume ventilation, but does not alter gross brain injury [39]. Brain injury can also be detected in the absence of gross neuropathology. One hour of injurious (high tidal volume) mechanical ventilation in preterm lambs (125–127 d gestation) results in changes of fractional anisotropy in the thalamus, but no anatomical or gross structural changes [30]. The absence of substantive MRI or anatomical pathology in the postnatal lambs suggests that relatively short-term (seven days) contemporary lung protective respiratory support may not be acutely damaging to brain development. Our lambs were born at 129 d gestation, using a best-practice volume-targeted lung-protective ventilation strategy with early extubation to non-invasive respiratory support. The efficacy of ventilation management is confirmed by the low incidence of gross pathology in our study, compared to the acutely ventilated groups observed in previous studies using lambs of similar gestations [39,40], despite the more extended duration of ventilator support in the current study.

Spontaneous lesions were evident in the frontal cortex, temporal lobe, occipital lobe, and deep to the parahippocampal gyrus of some lambs, particularly those in the naïve fetal control group. These lesions were characterized histologically by local red blood cell aggregates, reactive astrocytes, active microglia, myelin fragmentation and caspase 3 activation. The injuries we described are similar to reports of the hypertrophic reactive astrocytes and reactive microglia localized by MRI within white matter injury sites in fetal sheep exposed to hypoxia-ischemia [36]. The poor preservation of post-fixed tissue may affect MRI signals. It is possible that the use of long-term storage modified tissue quality and volumes [15], however, we are confident of our findings, as the lesions were confirmed with immunohistochemistry and there was no association with the duration of fixation and imaging quality or volumes. Spontaneous white matter lesions in unventilated control lambs (124–126 d gestation) were previously reported [39]. Nonetheless, the high prevalence of histopathology in the fetal control group was surprising and remains unexplained. 

## 5. Conclusions

Our methodology allows for the detection of subtle neuropathology and takes advantage of histological examination to confirm the nature of lesions observed in the imaged preterm lamb brains. The use of these novel techniques also provides a method to image and analyse archival tissue to gain new insights into neuropathology. High resolution MRI of fixed, ex vivo lamb brains, provides indices of brain volume with white and grey matter segmentation, and appearance suitable for examining focal lesions. 

MR imaging shows no obvious detrimental effects on preterm birth and early postnatal care using contemporary neonatal care, nor of postnatal dexamethasone treatment on the brain development of preterm lambs; nonetheless, group sizes were small and hence, we cannot exclude small effects. We also cannot exclude the possibility of neurological harm occurring from earlier gestation of exposure, when brain maturity is more comparable to preterm human infants, or effects observable at later developmental time points such as adolescence, where clinical findings are generally more robust.

MR imaging identified spontaneous lesions in brains of fetal and preterm lambs which cannot readily be observed by gross anatomical observation. The discrepancy in findings between gross anatomical observation and MR brain imaging highlights the value of using high-quality MRI scans to identify brain pathology in preclinical studies. The use of methods presented here indicates that there are opportunities to image and successfully analyse archival brain tissue, including from the preterm brain. 

## Figures and Tables

**Table 1:** Summary of preterm animal clinical data, and results of statistical tests.

	Fetal Group	Postnatal Groups		
	Naïve Control (*n* = 7)	Saline (*n* = 8)	Low-Dex (*n* = 9)	High-Dex (*n* = 8)
**Sex (female/male)**	3/4	5/3	2/7	3/5
**Birth weight (kg)**	3.99 ± 0.44 ***	2.94 ± 0.41	3.23 ± 0.40	2.99 ± 0.34
**Post-mortem weight (kg)**	3.99 ± 0.44 ****	2.84 ± 0.37	2.75 ± 0.29	2.69 ± 0.44
**Post-mortem brain weight (g)**	54.2 ± 6.85 *	47.4 ± 4.51	48.3 ± 4.04	47.5 ± 4.94
**Proportion of time on mechanical ventilation (%)**	NA	15.5 ± 12.53	16.6 ± 16.66	31.8 ± 29.60

Data are presented as mean ± SD, except for sex, which is presented as a ratio. Statistical comparisons used *t*-tests between Fetal Control and Saline groups, and 1-Way ANOVA for the preterm postnatal treatment group comparisons, except sex, which used Chi-square. Only the Naïve control vs. Saline group yielded statistically significant differences. Dex, dexamethasone. Significance: *t*-test, * *p* < 0.05, *** *p* < 0.001, **** *p* < 0.0001.

**Table 2:** Statistical analysis of volumetric measures obtained from MRI.

MRI Region		Effect of Prematurity	Effect of Postnatal Dexamethasone
Frontal Cortex	Total	^a^ t(13) = 1.071, *p* = 0.304	^d^ H(2) = 0.924, *p* = 0.630
	white matter	^a^ t(13) = 1.198, *p* = 0.252	^d^ H(2) = 1.366, *p* = 0.505
	grey matter	^a^ t(13) = 0.098, *p* = 0.923	^c^ F(2,22) = 0.829, *p* = 0.450
	Ratio white:grey	^a^ t(13) = 0.279, *p* = 0.785	^c^ F(2,22) = 1.527, *p* = 0.239
Hippocampus	Total	^b^ t(8.16) = 0.33, *p* = 0.750	^c^ F(2,22) = 0.003, *p* = 0.997

Dex, dexamethasone. Analysis of the effect of prematurity on MRI volumes compared Fetal Controls and Saline using standard *t*-tests (a) or Welch’s correction (b). Effect of postnatal dexamethasone compared the Saline, Low-Dex, and High-Dex groups using 1-way ANOVA (c) or Kruskal–Wallis ranks (d).

**Table 3:** Anatomical measures of preterm lamb brains.

	Fetal Group	Postnatal Groups		
	Naïve Control (*n* = 7)	Saline (*n* = 8)	Low-Dex (*n* = 9)	High-Dex (*n* = 8)
Anterior horn width (mm)	1.4 ± 0.1	1.8 ± 0.3	1.9 ± 0.3	1.6 ± 0.1
Hemisphere width (mm)	23.4 ± 0.4	22.6 ± 0.4	22.8 ± 0.7	23.0 ± 0.4
Rostro-caudal length (mm)	49.7 ± 1.1	47.9 ± 0.9	48.2 ± 0.7	46.4 ± 0.9
Hemi-cerebellar width (mm)	14.7 ± 1.1	12.4 ± 0.3	12.3 ± 0.6	13.4 ± 0.4
Frontal cortex thickness (mm)	1.3 ± 0.1	1.4 ± 0.0	1.4 ± 0.1	1.6 ± 0.1
Lateral cortex thickness (mm)	1.4 ± 0.1	1.4 ± 0.1	1.4 ± 0.1	1.4 ± 0.1

Data are presented as mean ± SEM. Statistical comparisons used *t*-tests between Fetal Control and Saline groups, and 1-Way ANOVA for the preterm postnatal treatment group comparisons. There were no statistically significant differences for any outcome measure (all *p* > 0.05). Dex, dexamethasone.

**Table 4:** Prevalence of pathological lesions on MRI confirmed using T1- and T2-weighted images.

	Fetal Group		Postnatal Groups					
	Naïve Control (*n* = 7)		Saline (*n* = 8)		Low-Dex (*n* = 9)		High-Dex (*n* = 8)	
Any Pathology	3	(42.9%)	1	(12.50%)	1	(11.1%)	0	(0%)
frontal	0	(0%)	1	(12.50%)	1	(11.1%)	0	(0%)
temporoparietal	1	(14.3%)	0	(0%)	0	(0%)	0	(0%)
occipital	3	(42.9%)	0	(0%)	0	(0%)	0	(0%)
Cystic dissolution	2	(28.6%)	0	(0%)	0	(0%)	0	(0%)
Immaturity (occipital)	1	(14.3%)	5	(62.5%)	2	(22.2%)	2	(25%)

Values are shown as *n* (%).

## Supplementary Materials

The following are available online at https://www.mdpi.com/2076-3425/10/4/211/s1. Supplementary Data S1: Detailed instructions on segmentation of cortical sub-regions, and cleaning of the segmentation data.
