# Supplementary material for: Ex Vivo MRI Analytical Methods and Brain Pathology in Preterm Lambs Treated with Postnatal Dexamethasone
Source: Brain Sci. 2020 Apr 3;10(4):211. doi: 10.3390/brainsci10040211 (PMC7226431; doi:10.3390/brainsci10040211)
Supplement: Supplementary file 1 [file brainsci-10-00211-s001.pdf]

Workflow of segmentation of the frontal cortex

Generating 3D Brain Volume

The following segmentation example was performed using 3D Slicer 4.8.0. In the segmentation editor we used "Create New Segmentation". We created a segment, and named it "mask" and using the "Threshold" tool to select only the brain, and "Apply as mask". This limited segmentation to only label voxels with intensity within the range set and prevents labelling of empty space. We created a segment to label the whole brain using the same threshold as above. The "Show 3D" button is used to generate a 3D volume of the MRI scan as the basis of further cortical segmentation based upon brain surface features. Using the "Islands" tool, and selecting "Keep largest island" to removes small imaging artefacts creating a brain render usable for segmentation (Figure 4A).

Creating Rough Frontal Cortex Segment

Creating a segment called "Frontal Cortex" we used the "Surface cut" tool to place fiducial markers on the 3D brain volume along the sulci and gyri (Figure 4B to D) defined in the atlas (Ella et al., 2017). Select as many fiducials as needed scattered on the brain surface and use the "Fill Inside" radio button, and apply (Figure 4E). It may be useful to perform several multiple small segments for small regions on the cortex surface.

Refining the Frontal Cortex Segment

The surface cut tool can result in small unlabelled regions near the cortex surface. Use the "Margin" tool and the "Grow" command to expand the volume, to fill in small gaps (Figure 4F). A 0.5 mm growth should be suitable. The frontal cortex volume generated (Figure 4G) needs refinement to remove non-cortical structure.

Refine the segmentation by using a combination of 3D painting of the 3D volume, and correction for non-cortical structures in the coronal and sagittal planes (Figure 4E). Ensure continuity of surface labelling to the cortex below to cover the whole gyrus fold in coronal plane. Removal of large deep brain structures such as the striatum can be assisted by using the "Surface cut" tool in the coronal slice view. The segmentation can be enhanced by use of "Smoothing".
